# On Tubercular Disease in the East

**Published:** 1855-07

**Authors:** 


ON TUBERCULAR DISEASE IN THE EAST. By DR. WILSON. (Indian Annals, No. 3, p. 182.)—In the Zillah jail at Rajshye, there was an average of 846 prisoners. The average annual mortality from tubercle was a little under 8 per 1000, or nearly the same rate as at home. Thus, in six years 165 men died, and in almost all post-mortem examinations were made. Tubercles were present in one fourth of the whole.

Dr. Wilson remarks:

"The natives of India form no exception to the dark races in other parts of the globe, or at least this much may be said, that exemption from phthisis is by no means so universal as has been supposed: if portions of the continent of India are so exempt it is very desirable to have information regarding them, as convincing as that given of places where the disease has been ascertained. It is not surprising that, previous to the universal practice of percussion and auscultation, the extent of the prevalence of the disease should have been overlooked, but at first sight it is surprising that it should not be universally recognized up to the present time; the explanation is found in the difficulty of always procuring post-mortem examinations, and in the peculiar and latent nature of the chest symptoms, and partly, no doubt, from education at home settling in the mind a belief to the contrary. * * *

"The disease, besides the common wasting form, which has procured for it the descriptive names of consumption and decline, shows itself in two varieties, more commonly than in Europe—the latent and febrile; the latter is, possibly, often only a hurried termination of the latent. In the latent form, the chest symptoms may never appear, the disease being fatal by hurrying on of concomitant disease in the bowels, the effect of climate being to excite to activity the abdominal symptoms." (p. 188.)

